# Cloperastine inhibits esophageal squamous cell carcinoma proliferation in vivo and in vitro by suppressing mitochondrial oxidative phosphorylation

**DOI:** 10.1038/s41420-021-00509-w

**Published:** 2021-06-21

**Authors:** Bo Li, Yin Yu, Yanan Jiang, Lili Zhao, Ang Li, Mingzhu Li, Baoyin Yuan, Jing Lu, Ziming Dong, Jimin Zhao, Kangdong Liu

**Affiliations:** 1 The Pathophysiology Department, School of Basic Medical Sciences, College of Medicine, Zhengzhou University, Zhengzhou, 450000 China; 2 China-US (Henan) Hormel Cancer Institute, Zhengzhou, 450000 China; 3 State Key Laboratory of Esophageal Cancer Prevention and Treatment, Zhengzhou, 450000 Henan China; 4 Provincial Cooperative Innovation Center for Cancer Chemoprevention, Zhengzhou University, 450000 Zhengzhou, Henan China; 5 Cancer Chemoprevention International Collaboration Laboratory, Zhengzhou, 450000 Henan China

**Keywords:** Cancer prevention, Cancer metabolism

## Abstract

Esophageal squamous cell carcinoma (ESCC) is a major type of esophageal cancer. The prognosis of patients with ESCC remains poor because of the high morbidity and mortality of the disease. One strategy for drug discovery for ESCC treatment or prevention is screening FDA-approved drugs. In the present study, we found that the antitussive agent cloperastine can inhibit the proliferation of ESCC cells. However, the underlying mechanism was unclear. To determine the mechanism of this inhibitory effect, we performed proteomic analysis using KYSE150 cells treated with cloperastine and DMSO. The results identified several down-regulated signaling pathways included those of three key proteins (NADH dehydrogenase [ubiquinone] 1 alpha subcomplex 1, NADH ubiquinone oxidoreductase subunit S5, and cytochrome C oxidase subunit 6B1) involved in oxidative phosphorylation. Meanwhile, we observed that oxidative phosphorylation in mitochondria was inhibited by the drug. Importantly, cloperastine suppressed ESCC growth in a xenograft mouse model in vivo. Our findings revealed that cloperastine inhibits the proliferation of ESCC in vivo and in vitro by suppressing mitochondrial oxidative phosphorylation.

## Introduction

Esophageal cancer is the sixth-leading cause of cancer death globally, and approximately 400,000 esophageal cancer patients died every year [1–3]. Esophageal squamous cell carcinoma (ESCC) comprises approximately 90% of esophageal cancer cases [4]. Although the clinical application of surgery, chemotherapy, and radiotherapy has improved patient prognosis, the 5-year survival rate remains less than 25% [3, 5, 6]. Thus, novel strategies are absolutely needed to reduce the mortality of patients with ESCC.

Mitochondrial metabolism plays an important role in the occurrence and development of tumors [7, 8]. Mitochondria represent the main source of ATP production, and they maintain the balance of reactive oxygen species (ROS) generation and elimination [9, 10]. Evidence indicates that targeting inhibition of mitochondrial metabolism suppresses the progression of multiple tumors [11–14]. Therefore, the role of mitochondrial metabolism in tumors increases the cancer researchers’ attention.

By screening a FDA-approved drug library, we found that cloperastine can significantly inhibit the proliferation of ESCC cells. Cloperastine is a drug with a central antitussive effect and an antihistamine effect [15]. Studies revealed that cloperastine acts on the cough center without suppressing the respiratory center, and it has been widely used in the clinic [16]. However, the function of cloperastine in tumor growth and development and its mechanism have not been clearly investigated.

In this study, we found that mitochondrial oxidative phosphorylation was inhibited after cloperastine treatment. Cloperastine also suppressed patient-derived esophageal xenograft tumor growth in vivo. Our work confirmed that cloperastine can inhibit ESCC cell proliferation and tumor growth by suppressing mitochondrial oxidative phosphorylation.

## Results

### Cloperastine inhibits ESCC cell proliferation

**Fig. 1:**
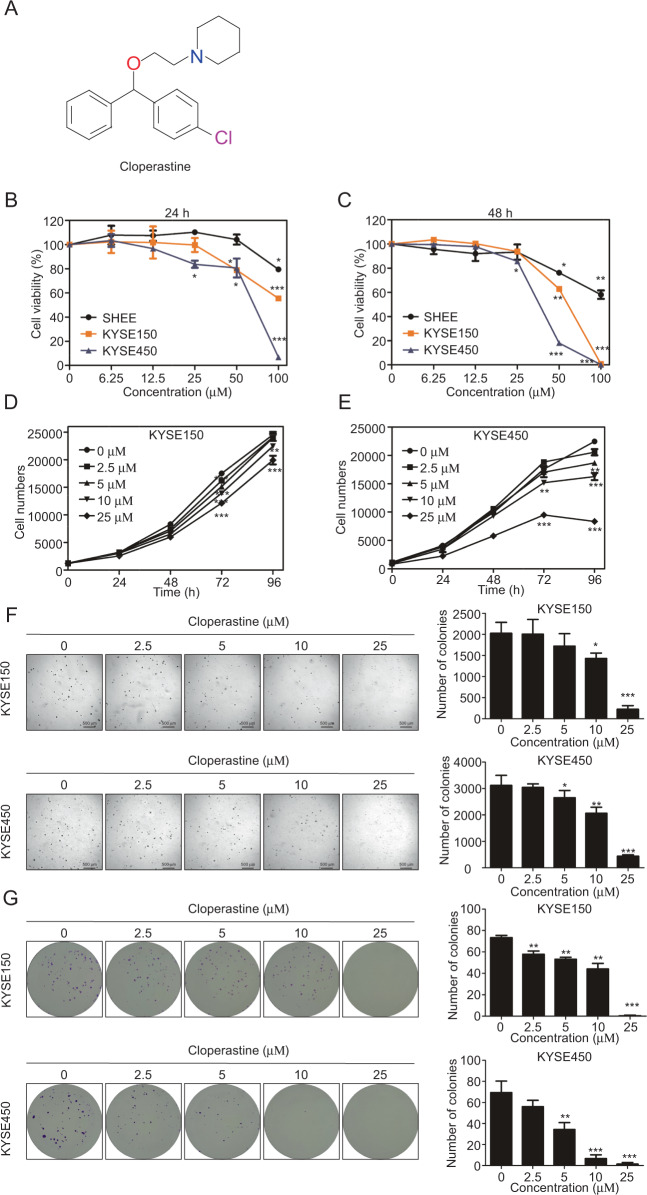
Cloperastine inhabit ESCC cell proliferation. **A** Chemical structure of cloperastine. **B**, **C** Cytotoxicity of cloperastine on SHEE, KYSE150, and KYSE450 cells. Cells were treated with cloperastine at various concentrations and then measured cell viability at 24 h and 48 h. **D**, **E** Effect of cloperastine on ESCC cell growth. Cells were treated with different doses of cloperastine (0, 2.5, 5, 10, 25 µM) and cells number was measured at 0, 24, 48, 72, 96 h. (*p < 0.05, **p < 0.01, ***p < 0.001). **F** Effect of cloperastine suppresses anchorage-independent growth of ESCC cells. KYSE150 and KYSE450 were treated with various concentration of cloperastine (0, 2.5, 5, 10, 25 μM) and measure at 7 days. (*p < 0.05, **p < 0.01, ***p < 0.001). **G** Effect of cloperastine suppresses colony formation of ESCC cells. KYSE150 and KYSE450 were treated with various concentration of cloperastine (0, 2.5, 5, 10, 25 μM) and measure at 12 days. (*p < 0.05, **p < 0.01, ***p < 0.001).

By screening a FDA-approved drug library, we revealed that cloperastine has cytotoxicity to KYSE450 cells (Supplementary Fig. 1A and Table 1). Cloperastine is an antitussive drug and is widely used to treat chronic cough in the clinic (Fig. 1A). To determine whether the cloperastine has cytotoxicity to normal esophageal epithelial or ESCC cells, cells were treated with various concentrations of cloperastine for 24 or 48 h. The results indicated that cloperastine had significantly stronger cytotoxicity in ESCC cells than on Shantou immortalized human esophageal (SHEE) cells (Fig. 1B, C). To determine the effects of cloperastine on ESCC cell proliferation, KYSE150 and KYSE450 cells were treated with various concentrations of cloperastine. The results demonstrated that cloperastine concentration-dependently inhibited cell growth (Fig. 1D, E). In addition, anchorage-independent growth was significantly inhibited by cloperastine in a dose-dependent manner (Fig. 1F). Meanwhile, a clone formation assay also illustrated that the number and size of clones were dose-dependently suppressed in the cloperastine treatment group compared to the control groups (Fig. 1G). These results revealed that cloperastine can inhibit ESCC cell proliferation in vitro.

**Table 1:** FDA-approved drug library.

Number	Name
1	Lidocaine hydrochloride hydrate
2	Naproxen sodium
3	Bergenin
4	Estradiol Dipropionate
5	Losartan
6	Metyrapone
7	Cyproheptadine hydrochloride
8	Amiloride hydrochloride dihydrate
9	Actarit
10	Cloperastine hydrochloride
11	Ranolazine dihydrochloride
12	Epinephrine Hydrochloride
13	2-Aminobenzenesulfonamide
14	Scopolamine hydrobromide trihydrate
15	Ropivacaine
16	Oxybutynin
17	Pioglitazone hydrochloride
18	Fluoxetine Hydrochloride
19	Glafenine Hydrochloride
20	Tacrine hydrochloride hydrate
21	Vardenafil
22	Ziprasidone HCl
23	Miconazole Nitrate
24	Amprolium Hydrochloride
25	Maltose monohydrate
26	Ferulic acid
27	Paeonol
28	Naringin
29	Sennoside A
30	Dirithromycin
31	Estrone
32	Isoniazid
33	Pyridoxine
34	Clevidipine
35	Yohimbine hydrochloride
36	Dorzolamide hydrochloride
37	Ethisterone
38	Novobiocin Sodium Salt
39	Triprolidine hydrochloride monohydrate
40	Cephalexin
41	Nifenazone
42	propantheline bromide
43	D-penicillamine
44	Oxacillin sodiuM salt
45	Amoxicillin
46	Carbenoxolone disodium,(3β,20β)-3-(3-Carboxy-1-oxopropoxy)-11-oxoolean-12-en-29-oicaciddisodium
47	2-amino-1-(2,5-dimethoxyphenyl)propan-1-ol,hydrochloride
48	Ketotifen fumarate
49	Chlorpyrifos
50	Tylosin
51	Trimetazidine dihydrochloride
52	Dicloxacillin sodium
53	Etodolac
54	Floxuridine

### Cloperastine suppresses NADH dehydrogenase (ubiquinone) 1 alpha subcomplex 1 (NDUFA1), NADH ubiquinone oxidoreductase subunit S5 (NDUFS5), and cytochrome C oxidase subunit 6B1 (COX6B1) expression

To explore the mechanism by which cloperastine inhibits ESCC cell proliferation, we performed proteomics using KYSE150 cells treated with DMSO or cloperastine (25 μM) for 24 h (Supplementary Fig. 2A). Subsequently, the differentially expressed proteins were screened via quality control analysis. In this study, we identified 6472 identified proteins, among which quantitative information was available for 5155 proteins, and the mass accuracy error of the mass spectrometer was within 10 ppm, which was in line with the high-precision characteristics of mass spectrometry. The results confirmed the mass accuracy of mass spectrometer, and the qualitative and quantitative analysis of the protein was not affected by excessive mass deviation (Supplementary Fig. 2B). In addition, most of the identified peptides were 7–20 amino acids in length, which conformed to the general rules based on trypsin enzymatic hydrolysis and high-energy collisional dissociation (HCD) fragmentation (Supplementary Fig. 2C). Altogether, quality control analysis confirmed that the test samples met the standards.

**Fig. 2:**
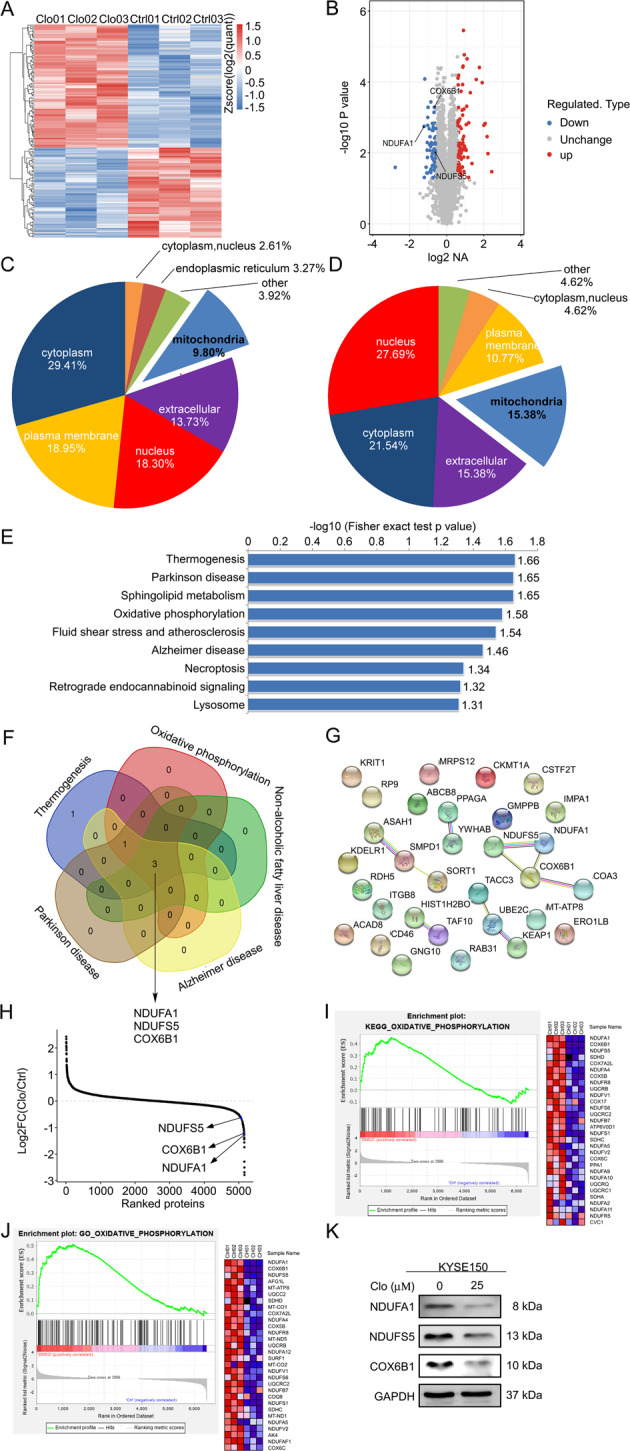
Cloperastine suppresses NADH dehydrogenase (ubiquinone) 1 alpha subcomplex 1 (NDUFA1), NADH ubiquinone oxidoreductase subunit S5 (NDUFS5), and cytochrome C oxidase subunit 6B1 (COX6B1) expression. **A** The heat map represents the differentially expressed protein in KYSE150 cells treated with DMSO or cloperastine for 24 h. **B** Volcano map shows 89 up-regulated proteins and 65 down-regulated proteins, and the positions of NDUFA1, NDUFS5, and COX6B1 are marked. **C** WoLF PSORT software analysis of the subcellular structure localization and classification analysis of differentially expressed proteins. **D** WoLF PSORT software analysis of the subcellular structure localization and classification analysis of down-regulated proteins. **E** Down-regulated KEGG pathways enrichment. Data were shown as −Log10 (Fisher’s exact test *p*-value). **F** Venn diagram shows a set of down-regulated five pathways and lists three down-regulated proteins, and NDUFA1, NDUFS5, and COX6B1 are marked. **G** Diagram of protein-protein interaction network of down-regulated proteins, and NDUFA1, NDUFS5, and COX6B1 are located. **H** The average quantitative kinase data is ranked according to its Log2 FC between the cells after DMSO and cloperastine, and shows the positions of NDUFA1, NDUFS5, and COX6B1. **I** GSEA analysis of the changes in oxidative phosphorylation of the KEGG gene set after DMSO and cloperastine treatment. **J** GSEA analysis of the changes in oxidative phosphorylation of the GO gene set after DMSO and cloperastine treatment. **K** Western blotting for NDUFA1, NDUFS5, and COX6B1 in KYSE150 cells after DMSO and cloperastine treatment.

After cloperastine treatment for 24 h, 154 differentially expressed proteins were detected in KYSE150 cells (Fig. 2A), including 89 up-regulated and 65 down-regulated proteins (Fig. 2B). We performed subcellular structure localization and classification analyses of the differentially expressed proteins. Interestingly, 9.8% of the up-regulated proteins and 15.38% of the down-regulated proteins were localized to the mitochondria (Fig. 2C, D). Therefore, we hypothesized that mitochondria may play an important role in the inhibitory effects of cloperastine on ESCC. Therefore, we examined differentially protein enriched in Kyoto Encyclopedia of Genes and Genomes (KEGG) pathways. Among the top nine down-regulated KEGG pathways, five pathways included three proteins: NDUFA1, NDUFS5, and COX6B1 (Fig. 2E, F). All three proteins are involved in the mitochondrial electron transport chain, and these proteins can interact directly (Fig. 2G). Meanwhile, the whole proteome results highlighted that NDUFA1, NDUFS5, and COX6B protein levels were significantly decreased after cloperastine treatment (Fig. 2H). Therefore, we used the Gene Set Enrichment Analysis database to analyze the proteomics results, uncovering that the expression of oxidative phosphorylation and electron transport chain proteins was decreased in both the KEGG and Gene Ontology databases after cloperastine treatment, and NFUFA1, NDUFS5, COX6B1 were the most strongly down-regulated proteins (Fig. 2I, J and Supplementary Fig. 2D). Subsequently, we used western blotting to verify the accuracy of the proteomics results, and the results illustrated that NFUFA1, NDUFS5, and COX6B1 protein expression was significantly decreased by cloperastine treatment (Fig. 2K), proving the reliability of the proteomics results. In short, proteomics revealed that cloperastine inhibited three key proteins (NDUFA1, NDUFS5, and COX6B1) involved in oxidative phosphorylation.

### Cloperastine suppresses oxidative phosphorylation

**Fig. 3:**
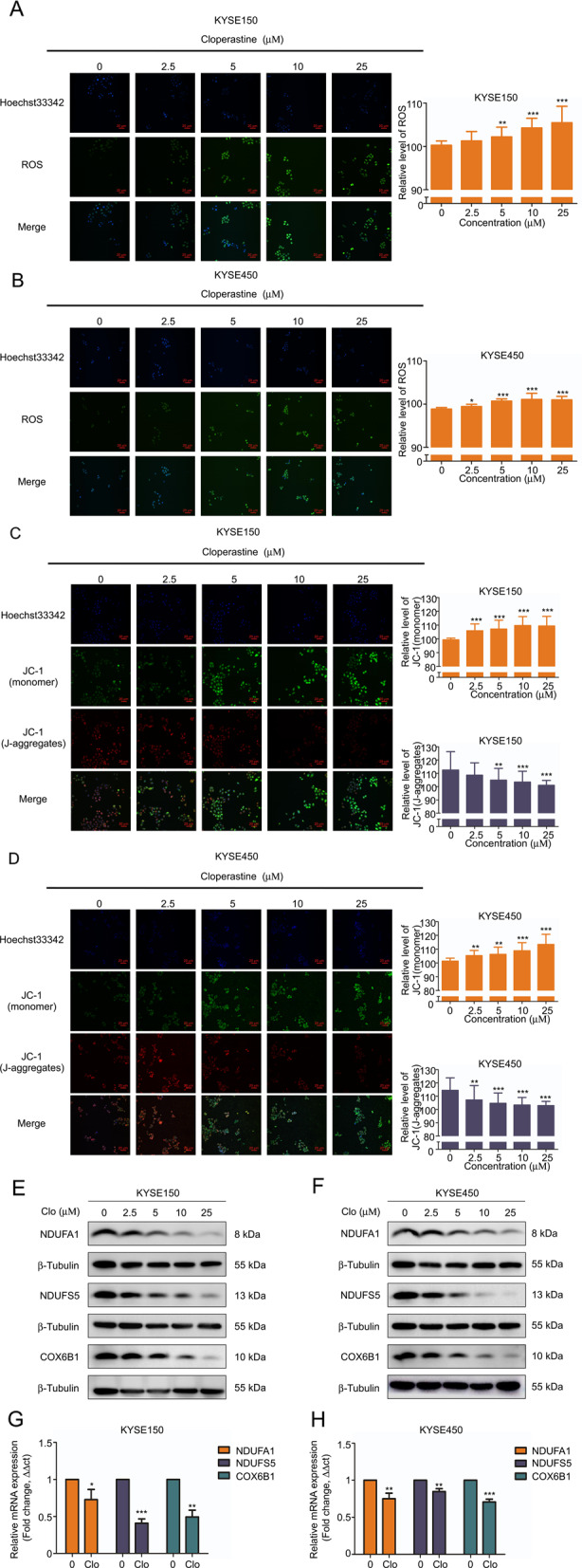
Cloperastine suppresses oxidative phosphorylation. **A**, **B** ROS levels of KYSE150 and KYSE450 after cloperastine treatment. Cells were treated with different doses of cloperastine (0, 2.5, 5, 10, 25 µM) and fluorescence intensity was measured at IN Cell Analyzer 6000. (*p < 0.05, **p < 0.01, ***p < 0.001) **C**, **D** JC-1 monomer and J-aggregate levels of cloperastine on KYSE150 and KYSE450 after cloperastine treatment. Cells were treated with different doses of cloperastine (0, 2.5, 5, 10, 25 µM) and fluorescence intensity was measured at IN Cell Analyzer 6000. (*p < 0.05, **p < 0.01, ***p < 0.001). **E**, **F** Protein’s level of NDUFA1, NDUFS5, and COX6B1 in KYSE150 and KYSE450 cells after treated with different doses of cloperastine (0, 2.5, 5, 10, 25 µM). **G**, **H** mRNA’s level of NDUFA1, NDUFS5, and COX6B1 in KYSE150 and KYSE450 cells after DMSO and cloperastine treatment.

NDUFA1, NDUFS5, and COX6B1 are the accessory subunits of mitochondrial oxidation respiratory chain complexes I and IV. Previous studies demonstrated that the downregulation of complex subunits leads to inhibition of the mitochondrial oxidative respiratory chain [17–20]. Uncoupling of the electron transport chain and oxidative phosphorylation in dysfunctional mitochondria leads to increased ROS production [21]. Therefore, we measured ROS levels in ESCC cells following cloperastine treatment. The results indicated that ROS levels were increased by cloperastine treatment (Fig. 3A, B and Supplementary Fig. 3A). The stability of mitochondrial membrane potential is an important index of the energy storage process during oxidative phosphorylation [10, 22]. We detected mitochondrial membrane potential using the JC-1 probe. The results revealed that the levels of JC-1 aggregates were decreased by cloperastine in a concentration-dependent manner, and whereas those of the monomer were concentration-dependently increased, meaning that mitochondrial membrane potential was decreased by increasing concentrations of cloperastine (Fig. 3C, D and Supplementary Fig. 3B). To investigate whether the NDUFA1, NDUFS5, and COX6B1 protein levels were related to the drug concentration, we confirmed by western blotting that the protein levels of NDUFA1, NDUFS5, and COX6B1 also decreased with increasing drug concentrations (Fig. 3E, F). At the same time, we observed that the mRNA expression of NDUFA1, NDUFS5, and COX6B1 was decreased by treatment (Fig. 3G, H), indicating that cloperastine inhibits NDUFA1, NDUFS5, and COX6B1 expression at the mRNA level. The results suggested that cloperastine inhibits the progression of ESCC by suppressing oxidative phosphorylation.

### The protein level of NDUFA1 is associated with the prognosis of patients with ESCC

**Fig. 4:**
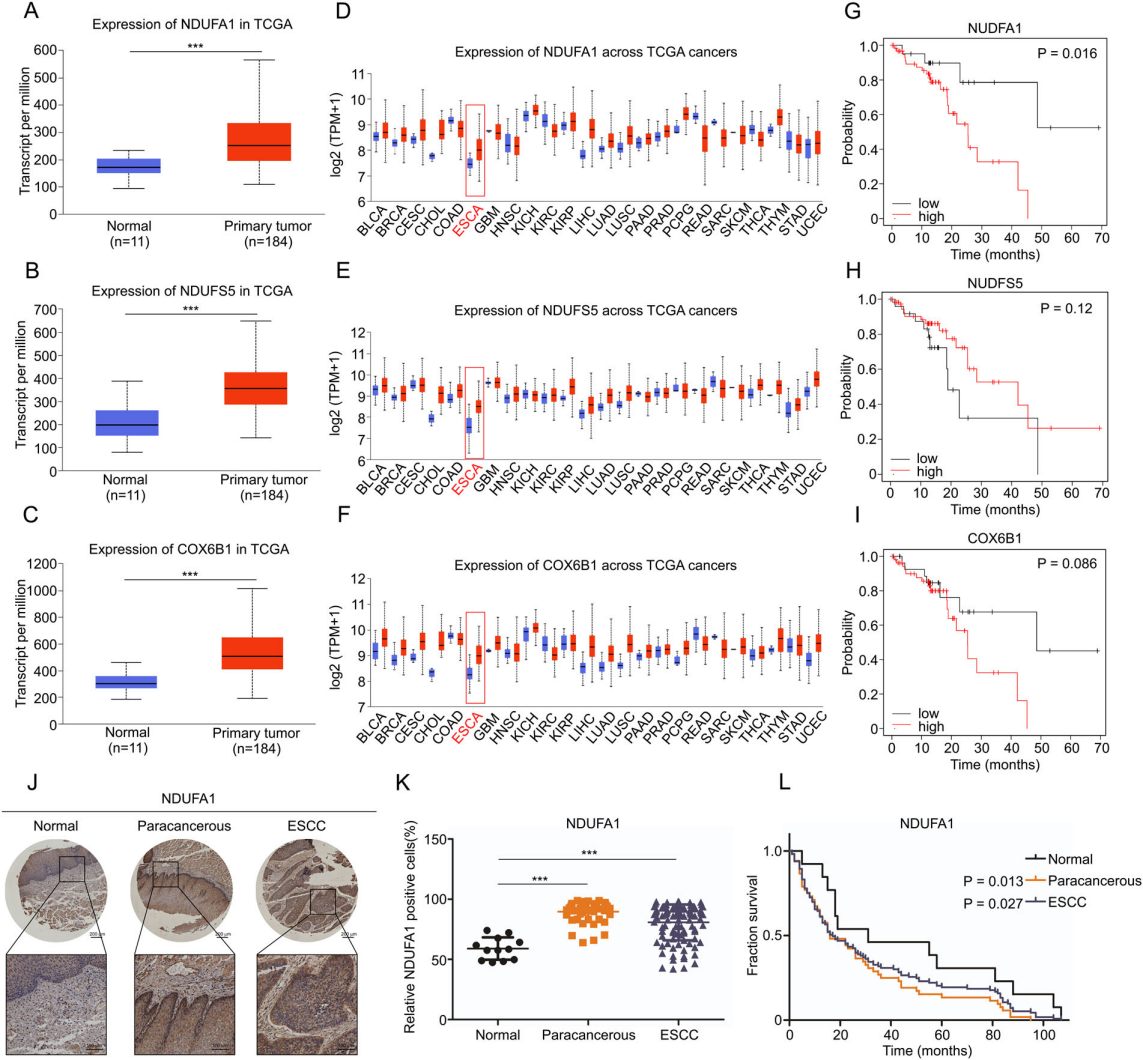
The protein level of NDUFA1 is associated with the prognosis of patients with ESCC. **A**–**C** TCGA database analyzes the expression of NDUFA1, NDUFS5, and COX6B1 in normal tissues and esophageal cancer tissues. **D**–**F** TCGA database analyzes the expression of NDUFA1, NDUFS5, and COX6B1 in normal tissues and different cancer tissues. **G**–**I** TCGA database analyzes the relationship between NDUFA1, NDUFS5, and COX6B1 and the prognosis of patients with esophageal cancer. **J**, **K** The level of NDUFA1 in normal tissues (*n* = 12), paracancerous tissues (*n* = 47), and ESCC tissues (*n* = 102). (*p < 0.05, **p < 0.01, ***p < 0.001). **L** Fraction survival of the level of NDUFA1 in normal tissues (*n* = 2), paracancerous tissues (*n* = 47), and ESCC tissues (*n* = 102) and *p*-value are marked.

Using The Cancer Genome Atlas database, we analyzed the relationships of NDUFA1, NDUFS5, and COX6B1 expression with esophageal cancer. The results demonstrated that the three proteins are all highly expressed in esophageal cancer (Fig. 4A–C). In addition, the differential expression was more prominent in pan-cancer (Fig. 4D–F). Among the three proteins, NDUFA1 is strongly related to the prognosis of esophageal cancer (Fig. 4G–I). Therefore, we analyzed NDUFA1 expression in normal esophageal epithelial, paracancerous, and ESCC tissues via tissue microarrays and found that NDUFA1 is highly expressed in paracancerous and ESCC tissues, and the difference was statistically significant (Fig. 4J, K). In addition, we analyzed survival in the three groups, and results indicate that survival was lower in the paracancerous and ESCC groups than in the normal group (Fig. 4L). These findings suggested that NDUFA1 may be a key protein associated with the poor prognosis of ESCC.

### Depletion of NDUFA1 inhibits ESCC cell proliferation

**Fig. 5:**
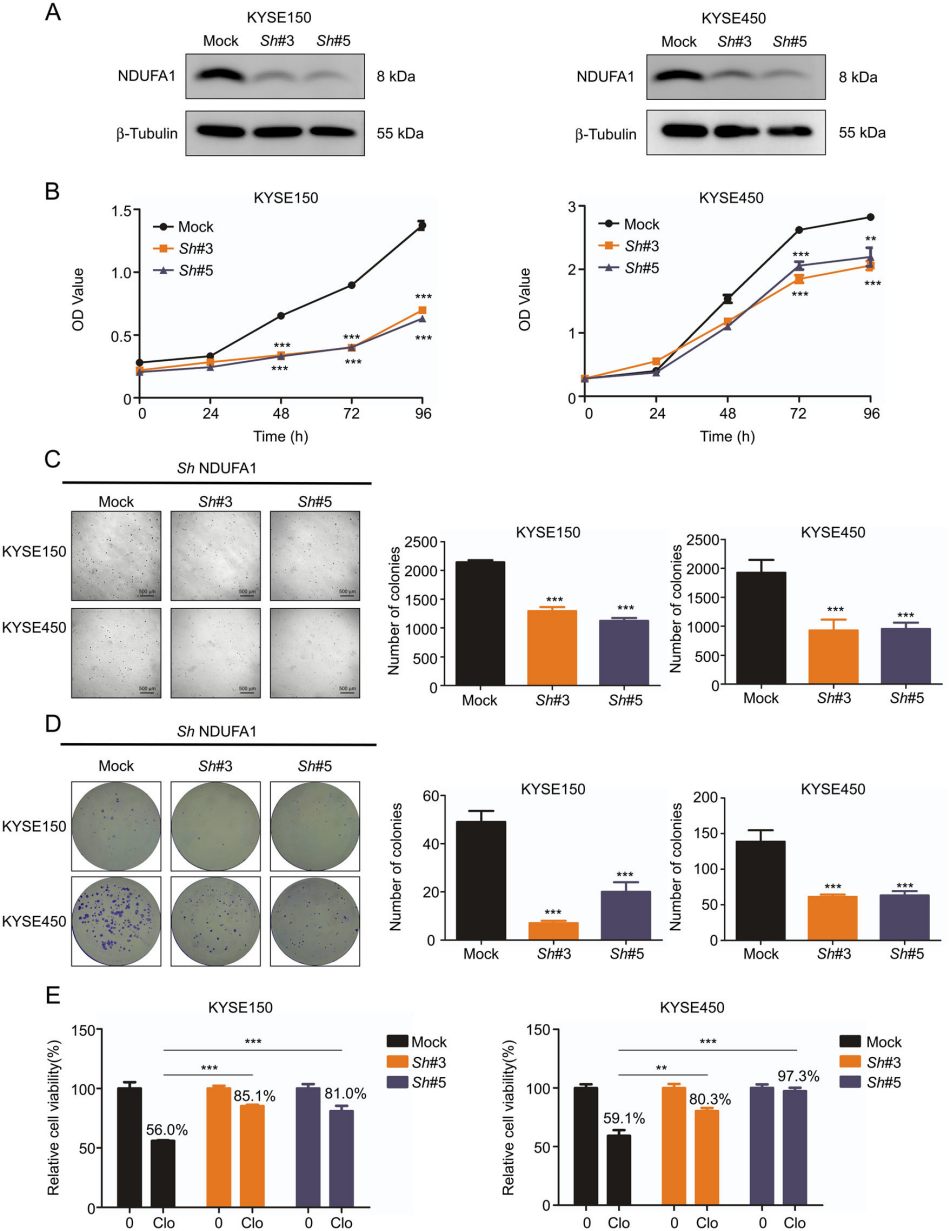
Depletion of NDUFA1 inhibits ESCC cell proliferation. **A** The short hairpin RNA (shNDUFA1) in the vector plko.1 was used to transduce KYSE150 and KYSE450 cells, and Western blotting detects the knockdown efficiency of selected shNDUFA1 in KYSE150 and KYSE450 cells. **B** Effect of shNDUFA1 in KYSE150 and KYSE450 cells cell growth. OD value was measured at 0, 24, 48, 72, 96 h by MTT assay. (*p < 0.05, **p < 0.01, ***p < 0.001). **C** Effect of shNDUFA1 in KYSE150 and KYSE450 cells anchorage-independent growth. Cells number was measured at 7 days. (*p < 0.05, **p < 0.01, ***p < 0.001). **D** Effect of shNDUFA1 in KYSE150 and KYSE450 cells colony formation. Cells number was measured at 12 days. (*p < 0.05, **p < 0.01, ***p < 0.001). **E** Effect of shNDUFA1 in KYSE150 and KYSE450 cells cell proliferation after cloperastine treatment. OD value was measured at 72 h by MTT assay. (*p < 0.05, **p < 0.01, ***p < 0.001).

Because NDUFA1 plays a role in ESCC patient prognosis, we depleted the gene in KYSE150 and KYSE450 cells (Fig. 5A) and assessed proliferation. NDUFA1 depletion resulted in decreases of proliferation, anchorage-independent growth, and colony formation in esophageal cancer cell lines (Fig. 5B–D), indicating that NDUFA1 plays an important role in cancer cell proliferation. In addition, the sensitivity of ESCC cells to cloperastine was decreased by NDUFA1 depletion (Fig. 5E), demonstrating that cloperastine inhibits ESCC cell proliferation partly through NDUFA1.

### Cloperastine inhibits patient-derived xenograft (PDX) tumor growth in vivo

**Fig. 6:**
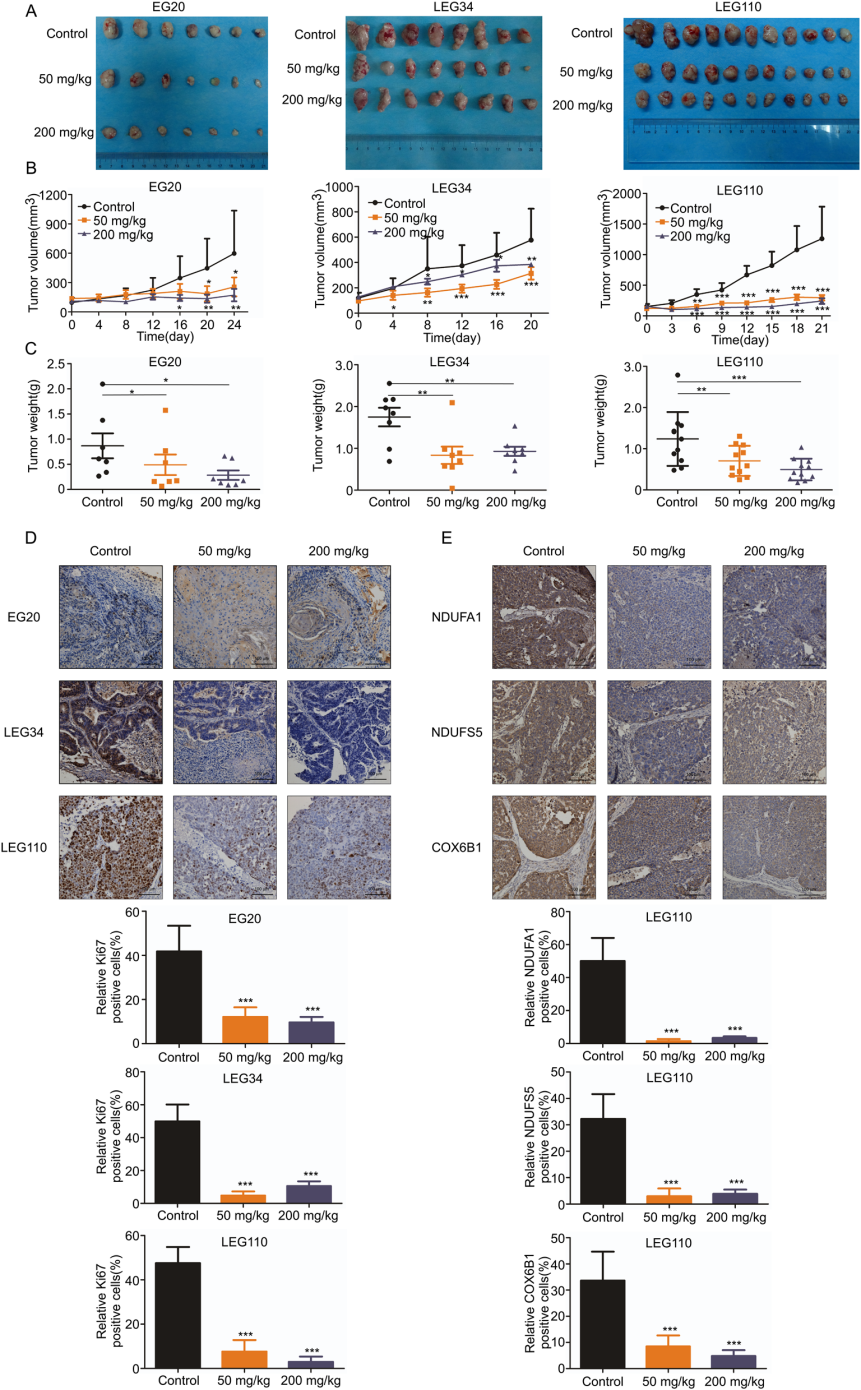
Cloperastine inhibits patient-derived xenograft (PDX) tumor growth in vivo. **A**–**C** Effect of cloperastine on ESCC tumor growth. Cloperastine significantly suppressed tumor growth of case EG20 (7 mice/group), LEG34 (8 mice/group) and LEG110 (11 mice/group). The tumor volume was measured every 3–5 days. (*p < 0.05, **p < 0.01, ***p < 0.001). **D** Immunohistochemical analysis the level of Ki67 in case EG20, LEG34, and LEG110 tumor tissues. The number of Ki67-stained cells was counted in down panel. (*p < 0.05, **p < 0.01, ***p < 0.001) (E) Immunohistochemical analysis (IHC) NDUFA1, NDUFS5, and COX6B1 protein levels in case LEG110 tumor tissues. The number of NDUFA1, NDUFS5, and COX6B1 stained cells was counted in down panel. (*p < 0.05, **p < 0.01, ***p < 0.001)Supplement Fig. 1 Screening drug from FDA-approved drug library. **A** Cytotoxicity of FDA-approved drugs on KYSE450 cells. Cells were treated with FDA-approved drugs at 50 μM and then measured cell viability at 48 h.Supplement Fig. 2 Mass spectrometry analysis based on proteome. **A** Flowchart for identification of quantitative proteomics. Control group treated with DMSO and the Treated group treated with cloperastine (25 μM) for 24 h in KYSE150 cells. **B** Peptide mass error. **C** The length distribution of peptides. **D** GSEA analysis of the changes in mitochondria respiratory electron transport of the KEGG gene set and in mitochondria aerobic electron transport chain of the GO gene set after DMSO and cloperastine treatment.Supplement Fig. 3 Cloperastine reduce oxidative phosphorylation. **A** ROS levels of KYSE150 and KYSE450 after cloperastine treatment. Cells were treated with DMSO or cloperastine (25 µM) and fluorescence intensity was observed using a laser-scanning confocal microscope (Olympus FV1000). **B** JC-1 monomer and J-aggregate levels of KYSE150 and KYSE450 after cloperastine treatment. Cells were treated with DMSO or cloperastine (25 µM) and fluorescence intensity was observed using a laser-scanning confocal microscope (Olympus FV1000). Supplement Fig. 4 Cloperastine inhibits patient-derived xenograft tumor growth of ESCC in vivo. **A** The tumor volume per mouse in case EG20, LEG34, and LEG110, the tumor volume was measured every 3–5 days. **B** Effect of cloperastine on mouse body weight of case EG20, LEG34, and LEG110. The body weight was measured every 2-3 days. **C** Tumor growth inhibition of case EG20, LEG34, and LEG110. (*p < 0.05, **p < 0.01, ***p < 0.001).

After confirming the inhibitory effect of cloperastine on ESCC cell growth in vitro, immunodeficient mice were used to establish a heterogeneous suppression model (PDX model) derived from patients with ESCC to test the effect of cloperastine in vivo [23, 24]. The tumor volume and weight were significantly reduced after treatment with cloperastine hydrochloride (50 or 200 mg/kg) compared to the findings in the control group (Fig. 6A–C and Supplementary Fig. 4A) without reducing body weight (Supplementary Fig. 4B). Subsequently, we performed immunohistochemical tests on the tumor tissues of the animals. Levels of the tumor proliferation biomarker Ki67 were significantly decreased in the treatment group (Fig. 6D). What’s more, we have observed that case LEG110 has a higher tumor growth inhibition compared to case EG20 and LEG34 (Supplementary Fig. 4C). Therefore, we have detected the expression of NDUFA1, NDUFS5, and COX6B1 in case LEG110, and the level of these three proteins was also decreased in the treatment group (Fig. 6E). The results indicated that cloperastine inhibited tumor growth was by suppressing NDUFA1, NDUFS5, and COX6B1 expression.

## Discussion

Chemoprevention by drugs can slow or reverse the development of cancer, thereby could reduce the incidence and mortality of cancer [25, 26]. FDA-approved drugs are valuable assets for finding effective chemoprevention drugs for ESCC. We used a cytotoxicity assay to screen an FDA-approved drug library and found that cloperastine is cytotoxic to KYSE450 ESCC cells. In the present study, cloperastine significantly inhibited ESCC cell growth and patient-derived xenograft tumor growth in vivo (Fig. 1, 6). We have demonstrated for the first time that cloperastine can inhibit ESCC cell proliferation in vitro and in vivo.

Mitochondrial metabolism plays a key role in tumor progression [8, 27]. Mitochondrial metabolism has basic bioenergy functions and provides an appropriate basis for tumor anabolism, the redox balance, and cell death [9, 13]. In this study, we found that the expression of the complex I and IV subunits NDUFA1, NDUFS5, and COX6B1, which are involved in oxidative phosphorylation, were significantly decreased after treatment (Fig. 2), resulting in the inhibition of oxidative phosphorylation (Fig. 3). It has been reported that certain cancers, including ESCC, acute lymphoblastic leukemia, non-Hodgkin’s lymphoma, endometrial cancer, colorectal cancer, ovarian cancer, prostate cancer, head and neck cancer, lung adenocarcinoma, and thyroid cancer strongly rely on oxidative phosphorylation [14, 28, 29]. The findings indicated that oxidative phosphorylation is a potential target for cancer treatment. We also observed that NDUFA1 expression is elevated in esophageal cancer, and lower NDUFA1 expression is correlated with better patient prognosis (Fig. 4). NDUFA1 is an important subunit of mitochondrial oxidative respiratory chain complex I. Previous studies illustrated that mutations in the NDUFA1 gene lead to loss of function of complex I, which further inhibits the occurrence of oxidative phosphorylation [17]. In this study, we found that the proliferation and clone formation of esophageal cancer cells were significantly inhibited after NDUFA1 depleted (Fig. 5). We confirmed that cloperastine inhibited the proliferation of ESCC cells by suppressing oxidative phosphorylation. On the basis of the aforementioned results, we further investigated the effects of the drug in vivo using an ESCC PDX model. The results supported that cloperastine suppressed ESCC growth by reducing oxidative phosphorylation (Fig. 6).

In conclusion, our study revealed that cloperastine inhibits ESCC progression in vitro and in vivo. Our research results further indicated that cloperastine may be a potential chemo preventive drug for esophageal cancer.

## Methods

### Cell culture

KYSE150 and KYSE450 ESCC cells were purchased from the Chinese Academy of Sciences Cell Bank (Shanghai, China). SHEE cells were a gift from Shantou University. All the cell lines were mycoplasma-free and authenticated by STR analysis. KYSE150 and KYSE450 cells were cultured in RPMI 1640 medium, and SHEE cells were cultured in DMEM containing 10% FBS at 37 °C in an atmosphere of 5% CO_2_. All experiments were completed in triplicate to confirm the reliability of the results and obtain appropriate statistical analysis capabilities.

### Reagents and antibodies

Cloperastine was purchased from Nantong Feiyu Biological Technology (Nantong, China). Antibodies against NDUFA1, NDUFS5, COX6B1, and Ki67 were purchased from Abcam (Cambridge, UK). Hoechst 33342 and an oxygen species (ROS) assay kit were purchased from Solarbio Life Sciences (Beijing, China).

### Cytotoxicity assay

SHEE (8000 cells/well), KYSE150 (8000 cells/well), and KYSE450 cells (12000 cells/well) were seeded in 96-well plates and treated with various concentrations of cloperastine (0, 6.25, 12.5, 25, 50, and 100 μM). After 48 h, the cells were fixed, and the nuclei were stained with DAPI and then counted using IN Cell Analyzer 6000 software.

### Cell proliferation assay

KYSE150 (3000 cells/well) and KYSE450 cells (5000 cells/well) were seeded in 96-well plates and treated with various concentrations of cloperastine (0, 2.5, 5, 10, and 25 μM) for 0, 24, 48, 72, and 96 h. After treatment, the cells were fixed, and the nuclei were stained with DAPI and then counted using IN Cell Analyzer 6000 software.

### Anchorage-independent cell growth

KYSE150 and KYSE450 cells (8000 cells/well) were seeded in 10% FBS and 0.3% agar in the top gel, and different concentrations of cloperastine (0, 2.5, 5, 10, and 25 μM) were added to the mixed agar top gel and bottom gel. After 2 h of incubation at room temperature, the culture was placed in a 37 °C, 5% CO_2_ incubator for 1 week, and then the clones were counted and photographed using IN Cell Analyzer 6000 software.

### Colony formation assay

KYSE150 and KYSE450 cells (200 cells/well) were inoculated in six-well plates and treated with different concentrations of cloperastine (0, 2.5, 5, 10, and 25 μM). The medium was changed every 3 days, and crystal violet staining was performed after 10 days. After 3 min of staining, the clones were photographed and counted.

### Cell sample preparation and proteomic analysis

KYSE150 cells (4.5 × 10^6^) were evenly dispersed in a 15-cm dish and treated with 25 μM cloperastine for 24 h. Cells were subsequently collected for protein extraction. The sample was passed through a high-pH fractionation Agilent 300 Extend C18 column for reversed-phase HPLC (5-μm particles, 4.6 mm, 250 mm length). Briefly, the peptides were first separated into 60 fractions over 60 min using an acetonitrile (pH 9.0) gradient of 8%–32%. Then, the peptides were combined into six separate parts and dried via vacuum centrifugation. The peptides were subjected to NSI sources followed by tandem mass spectrometry (MS/MS) in the online connection of Q Exactive™ Plus (Thermo Fisher Scientific) to UPLC. Data obtained by searching the database were used to identify peptides assembled into proteins. The MS/MS data (v.1.5.2.8) were processed using the Maxquant search engine and then analyzed.

### ROS assay

KYSE150 and KYSE450 cells were inoculated in 96-well plates. After 16 h, cells were treated with various concentrations of cloperastine (0, 2.5, 5, 10, and 25 μM) for 24 h. DCFH-DA was diluted in fresh medium (without FBS) to a final concentration of 10 mM. After incubation with the diluted medium, the liquid was removed, and cells were washed twice with PBS. Then, 100 μL of diluted DCFH-DA (10 μM) were added to each well in the plate. Cells were incubated in a 37 °C incubator for 20 min [30]. After three washes with fresh medium (without FBS), the ROS content in the cells was measured using IN Cell Analyzer 6000 software.

### Mitochondrial membrane potential assay

KYSE150 and KYSE450 cells were inoculated in 96-well plates. After 16 h, cells were treated with various concentrations of cloperastine (0, 2.5, 5, 10, and 25 μM) for 24 h. JC-1 was diluted in fresh medium (without FBS) to a final concentration of 10 mM. After incubation, the medium was removed, and cells were washed twice with PBS. Then, 100 μL of diluted JC-1 (10 μM) were added to each well in the plate [31, 32]. Cells were incubated in a 37 °C incubator for 30 min. After three washes with fresh medium (without FBS), the levels of JC-1 monomers and aggregates in the cells were measured using IN Cell Analyzer 6000 software.

### Laser-scanning confocal microscopy

KYSE150 and KYSE450 cells were inoculated in a special confocal dish at 60 000 cells per dish. After 16 h, cells were treated with DMSO or cloperastine (25 μM) for 24 h. The levels of ROS and JC-1 monomers and aggregates were observed using a laser-scanning confocal microscope (Olympus FV1000).

### Western blotting

A protein assay kit (BCA Protein Assay Kit, Beyotime, China) was used to determine the concentration of the collected protein. Equal amounts of protein were separated using SDS-PAGE gels, and the separated proteins were transferred to a PVDF membrane. The membrane was blocked with 5% skimmed milk for 2 h, incubated with antibodies overnight at 4 °C, and then incubated with appropriate secondary antibodies linked to horseradish peroxidase at room temperature for 2 h. Enhanced chemiluminescence detection reagents were employed to visualize protein bands.

### MTT assay

KYSE150 (3000 cells/well) and KYSE450 cells (5000 cells/well) were seeded into 96-well plates and incubated for 0, 24, 48, 72, or 96 h. MTT (5 mg/mL, 10 μL/well) was added to each well, and the plate was incubated at 37 °C for 2 h. Cell proliferation was measured using a spectrometer.

### Plasmid construction, transfection, and lentivirus transduction

KYSE150 and KYSE450 cells were transfected with short hairpin RNA (shNDUFA1). The shNDUFA1 plasmid was cloned into the lentiviral expression vector plko.1. The full hairpin sequences of human shNDUFA1 were as follows: sh3, 5′-TGGGTATCACTGGAGTCTGAT-3′; and sh5, 5′-GCATCTCTGGAGTTGATCGTT-3′. Transfection efficiency was verified via western blotting.

### PDX mouse model

SCID/CB17 mice aged 5–6 weeks were purchased from Beijing Vital River Laboratory Animal Technology (Beijing, China). All animal experiments performed in this study were approved by the Zhengzhou University Institutional Animal Care and Use Committee. First, the mice were anesthetized with 0.4% sodium pentobarbital. Tumor tissues were cut into small pieces of 10–15 mm^3^ and implanted under the skin of the back of the neck of each mouse using forceps. After approximately 3–5 days, at which time the wound on the neck and back of the mouse had healed, the tumor volume of the mouse was measured at regular intervals. When the tumor volume reached 1000 mm^3^, the mouse was sacrificed, and the tumor tissue was excised. The removed tumors were processed and implanted into new SCID/CB17 mice in the same manner. When the transplanted tumor was stably passed to the third generation, the PDX mouse model of esophageal cancer had been successfully established.

ESCC PDX model mice were divided into 3 groups according to the random number table method. Groups are as follows: (1) Control group. (2) Low treatment group was gavage with 50 mg/kg cloperastine hydrochloride every day. (3) High treatment group were gavage with 200 mg/kg cloperastine hydrochloride every day.

### Immunohistochemical analysis

Formalin-fixed tumor tissue was embedded in paraffin, cut into 4-μm sections, and placed on a glass slide. The sample was baked in a constant temperature oven at 65 °C for 2 h, dewaxed, rehydrated, and then subjected to antigen retrieval. Next, H_2_O_2_ was dropped onto the tissue, followed by incubation at room temperature for 10 min to inactivate endogenous peroxidase. Then, the tissue was incubated with the prepared primary antibody solution at 4 °C overnight. All tissue slides were washed three times and incubated with HRP-IgG secondary incubation antibody at 37 °C for 15 min. DAB staining solution and hematoxylin staining solution were added dropwise for 1 min for counterstaining, and each slide was covered with cover glass after dehydration.

### Statistical analysis

Significant differences were compared using one-way analysis of variance or non-parametric tests, and the quantitative results were expressed as the mean ± SD. *p* < 0.05 was considered statistically significant. The Social Science Statistical Package (IBM, Inc. Armonk, NY, USA.) was used for statistical analysis.

## Supplementary information

Supplement Figure 1

Supplement Figure 2

Supplement Figure 3

Supplement Figure 4

## Data Availability

The datasets used and/or analyzed during the current study are available from the corresponding author on reasonable request.
