# Supplementary figures and images for: Cloperastine inhibits esophageal squamous cell carcinoma proliferation in vivo and in vitro by suppressing mitochondrial oxidative phosphorylation
Source: Cell Death Discov. 2021 Jun 21;7:166. doi: 10.1038/s41420-021-00509-w (PMC8257628; doi:10.1038/s41420-021-00509-w)

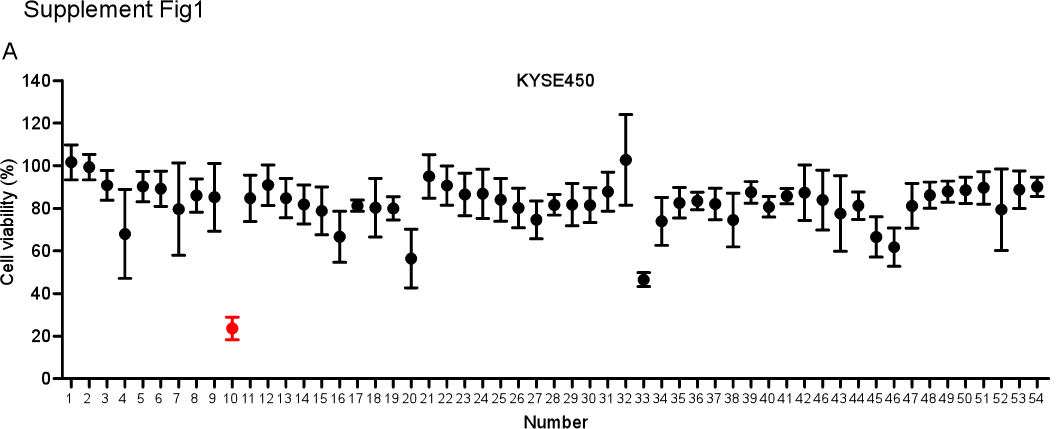

Supplement: Supplementary file 1 — Supplement Figure 1 [file 41420_2021_509_MOESM1_ESM.tif]

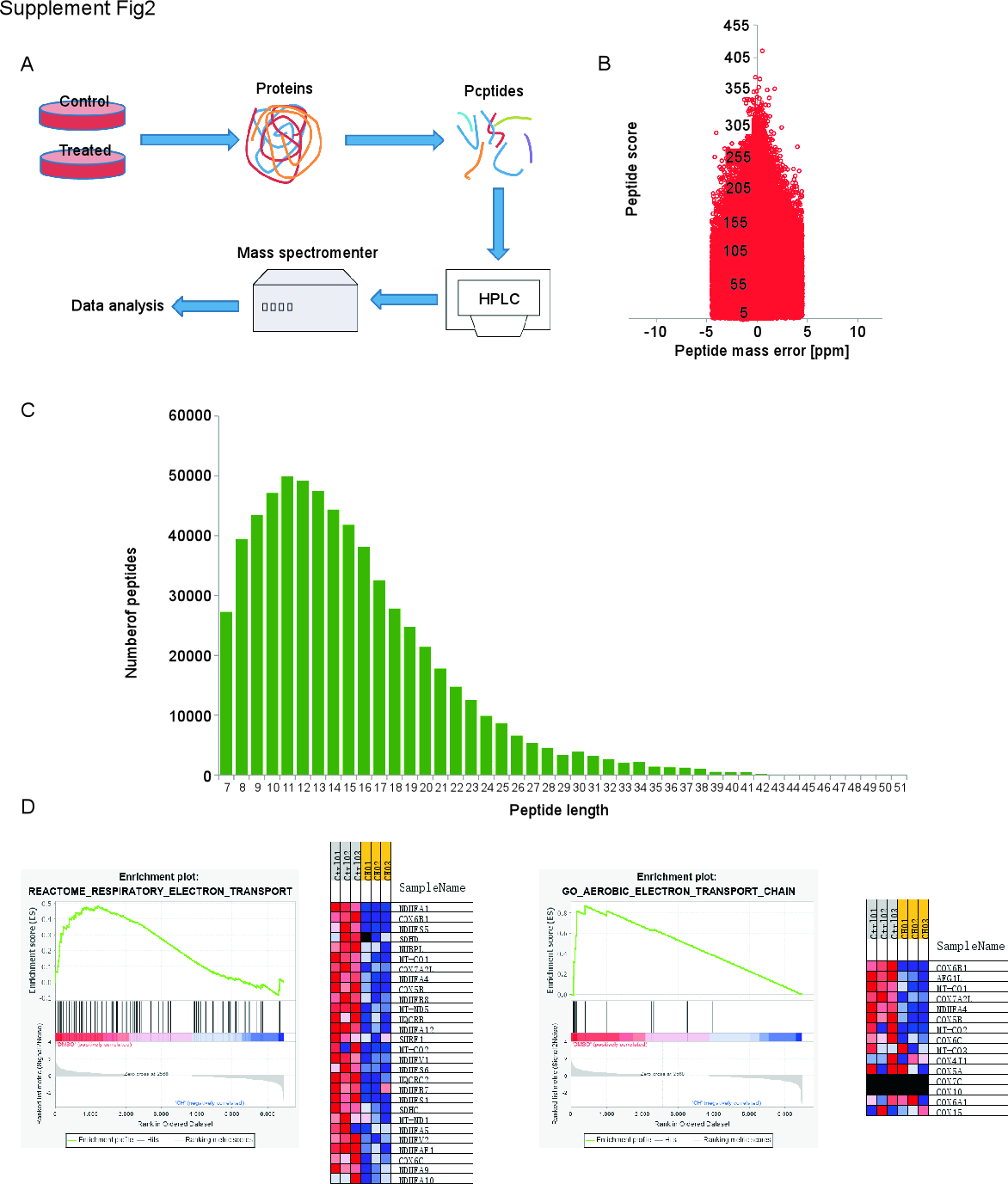

Supplement: Supplementary file 2 — Supplement Figure 2 [file 41420_2021_509_MOESM2_ESM.tif]

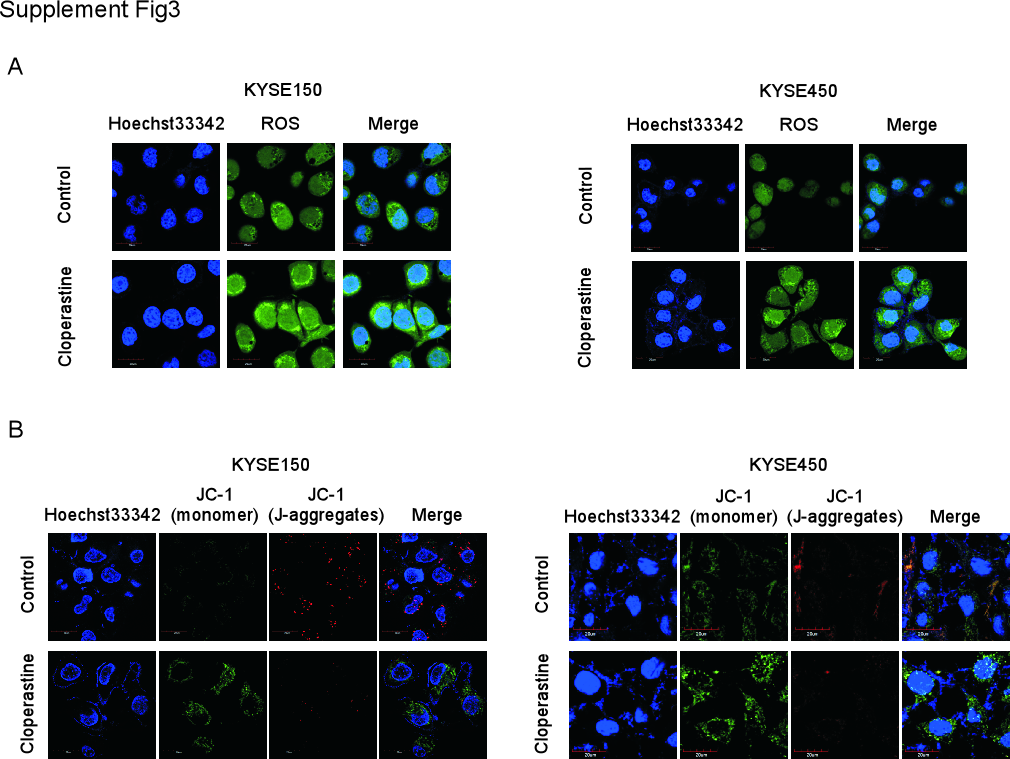

Supplement: Supplementary file 3 — Supplement Figure 3 [file 41420_2021_509_MOESM3_ESM.tif]

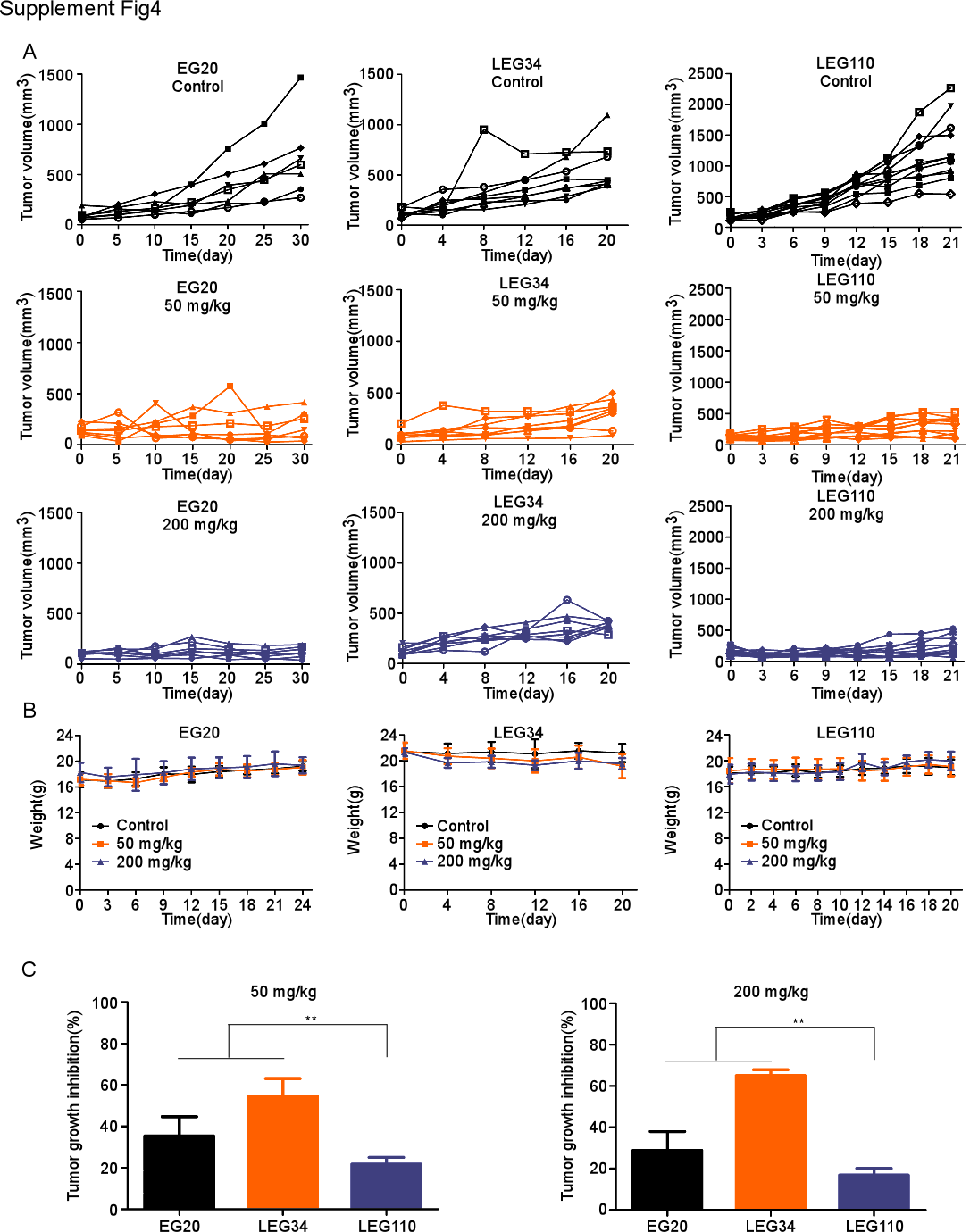

Supplement: Supplementary file 4 — Supplement Figure 4 [file 41420_2021_509_MOESM4_ESM.tif]
